# Supplementary material for: Effect of Nordic walking on walking ability in patients with peripheral arterial disease: a meta-analysis
Source: PLoS One. 2025 Mar 10;20(3):e0316092. doi: 10.1371/journal.pone.0316092 (PMC11892863; doi:10.1371/journal.pone.0316092)
Supplement: S1 Data — (DOCX) [file pone.0316092.s002.docx]

**Supplementary Table 3：Maximum walking distance（MWD） assessed by a treadmill test for PAD patients treated by Nordic walking programs in comparison to supervised exercise therapy.**

|  |  | **Nordic Walking program** | | | | | | **supervised exercise therapy** | | | | | |
| --- | --- | --- | --- | --- | --- | --- | --- | --- | --- | --- | --- | --- | --- |
| **ID** | **Study** | **Baseline**  **N** | **Post-intervention**  **N** | **Baseline** | **4-6weeks** | **12weeks** | **24weeks** | **Baseline**  **N** | **Post-intervention**  **N** | **Baseline** | **4-6weeks** | **12weeks** | **24weeks** |
| NO.1 | Collins 2012 [23] | 51 | 34 | 357±140 | 475±394 | 612±460 | 726±596 | 52 | 43 | 386±174 | 681±503 | 889±741 | 1020±825 |
| NO.2 | Girold 2017 [12] | 9 | 9 | 568±268 | 825±288 | NR | NR | 8 | 8 | 735±265 | 993±309 | NR | NR |
| NO.3 | Bulinska 2016 [29] | 21 | 21 | 224±144 | NR | 364±225 | NR | 31 | 31 | 164±75 | NR | 290±222 | NR |
| NO.4 | Kropielnicka 2018 [27] | 32 | 21 | 235±147 | NR | 373±232 | NR | 32 | 31 | 166±72 | NR | 303±229 | NR |

NR=not reported.

Data in the table are the mean ± SD (rounded to the nearest whole number) of the maximum walking distances in meters in the treadmill test.

**Supplementary Table 4：Maximum walking distance（MWD） assessed by a 6-minute walk test (6-MWT) for PAD patients treated by Nordic walking programs in comparison to supervised exercise therapy.**

|  |  | **Nordic Walking program** | | | | | **supervised exercise therapy** | | | | |
| --- | --- | --- | --- | --- | --- | --- | --- | --- | --- | --- | --- |
| **ID** | **Study** | **Baseline**  **N** | **Post-intervention**  **N** | **Baseline** | **4-6weeks** | **12weeks** | **Baseline**  **N** | **Post-intervention**  **N** | **Baseline** | **4-6weeks** | **12weeks** |
| NO.1 | Girold 2017 [12] | 9 | 9 | 477±139 | 546±131 | NR | 8 | 8 | 498±55 | 504±58 | NR |
| NO.2 | Bulinska 2016 [29] | 21 | 21 | 354±56 | 366±60 | 393±64 | 31 | 31 | 344±66 | 350±74 | 375±70 |
| NO.3 | Dziubek 2020 [28] | 32 | 21 | 354±56 | NR | 393±64 | 32 | 31 | 344±66 | NR | 375±70 |
| NO.4 | Kropielnicka 2018 [27] | 32 | 21 | 357±59 | NR | 391±66 | 32 | 31 | 344±53 | NR | 381±63 |

NR=not reported.

Data in the table are the mean ± SD (rounded to the nearest whole number) of the maximum walking distances in meters in the 6-MWT.

**Supplementary Table 5：Claudication distance (CD) assessed by a treadmill test for PAD patients treated by Nordic walking programs in comparison to supervised exercise therapy.**

|  |  | **Nordic Walking program** | | | | | | **supervised exercise therapy** | | | | | |
| --- | --- | --- | --- | --- | --- | --- | --- | --- | --- | --- | --- | --- | --- |
| **ID** | **Study** | **Baseline**  **N** | **Post-intervention**  **N** | **Baseline** | **4-6weeks** | **12weeks** | **24weeks** | **Baseline**  **N** | **Post-intervention**  **N** | **Baseline** | **4-6weeks** | **12weeks** | **24weeks** |
| NO.1 | Collins 2012 [23] | 51 | 34 | 179±97 | 184±121 | 251±242 | 449±584 | 52 | 43 | 203±130 | 227±193 | 213±121 | 328±232 |
| NO.2 | Bulinska 2016 [29] | 21 | 21 | 115±92 | NR | 166±98 | NR | 31 | 31 | 89±35 | NR | 151±95 | NR |
| NO.3 | Kropielnicka 2018 [27] | 32 | 31 | 118±97 | NR | 169±98 | NR | 32 | 21 | 94±37 | NR | 157±97 | NR |

NR=not reported.

The data in the table are the mean ± SD (rounded to the nearest whole number) of the claudication distances in meters in the treadmill test

**Supplementary Table 6：Claudication distance (CD) assessed by a a 6-minute walk test (6-MWT) for PAD patients treated by Nordic walking programs in comparison to supervised exercise therapy.**

|  |  | **Nordic Walking program** | | | | | | **supervised exercise therapy** | | | | | |
| --- | --- | --- | --- | --- | --- | --- | --- | --- | --- | --- | --- | --- | --- |
| **ID** | **Study** | **Baseline**  **N** | **Post-intervention**  **N** | **Baseline** | **4-6weeks** | **12weeks** | **24weeks** | **Baseline**  **N** | **Post-intervention**  **N** | **Baseline** | **4-6weeks** | **12weeks** | **24weeks** |
| NO.1 | Bulinska 2016 [29] | 21 | 21 | 163±91 | 230±90 | 237±94 | NR | 31 | 31 | 220±96 | 250±112 | 256±103 | NR |
| NO.2 | Dziubek 2020 [28] | 32 | 21 | 149±92 | NR | 182±103 | NR | 32 | 31 | 167±111 | NR | 182±123 | NR |

NR=not reported.

The data in the table are the mean ± SD (rounded to the nearest whole number) of the claudication distances in meters in the 6-MWT

**Supplementary Table 7：exercise duration assessed by a treadmill test for PAD patients treated by Nordic walking programs in comparison to supervised exercise therapy.**

|  |  | **Nordic Walking program** | | | | | | **supervised exercise therapy** | | | | | |
| --- | --- | --- | --- | --- | --- | --- | --- | --- | --- | --- | --- | --- | --- |
| **ID** | **Study** | **Baseline**  **N** | **Post-intervention**  **N** | **Baseline** | **4-6weeks** | **12weeks** | **24weeks** | **Baseline**  **N** | **Post-intervention**  **N** | **Baseline** | **4-6weeks** | **12weeks** | **24weeks** |
| NO.1 | Collins 2012 [23] | 51 | 34 | 7.30±2.89 | 9.83±8.16 | 12.67± 9.52 | 15.02± 12.32 | 52 | 43 | 7.98±3.60 | 14.09±10.40 | 18.39±15.34 | 21.10±17.07 |
| NO.2 | Collins（duration）2012 [25] | 45 | 35 | 8.60±3.92 | NR | 10.83±3.77 | NR | 40 | 36 | 8.35±4.22 | NA | 12.31±4.27 | NA |

NR=not reported.

The data in the table are the mean ± SD (rounded to the nearest whole number) of the exercise duration in meters in the treadmill test.

**Supplementary Table 8：Maximum walking distance（MWD） assessed for PAD patients treated by Nordic walking programs in comparison to control groups.**

|  |  | **Nordic Walking program** | | | | | | **Control group** | | | | | |
| --- | --- | --- | --- | --- | --- | --- | --- | --- | --- | --- | --- | --- | --- |
| **ID** | **Study** | **Baseline**  **N** | **Post-intervention**  **N** | **Baseline** | **4-6weeks** | **12weeks** | **24weeks** | **Baseline**  **N** | **Post-intervention**  **N** | **Baseline** | **4-6weeks** | **12weeks** | **24weeks** |
| NO.1 | Langbein 2002 [24] | 27 | NR | 488±418 | 927±813 | 1001±913 | 1373±931 | 25 | NR | 514±422 | 502±289 | 541±420 | 527±570 |
| NO.2 | Spafford 2014 [26] | 28 | 19 | 453±225 | NR | 652±373 | NR | 24 | 19 | 353±130 | NR | 382±186 | NR |

NR=not reported. The control group of Langbein et al. [24] received non routine exercise therapy, while the control group of Spafford et al. [26] received standard home exercise therapy.

**Supplementary Table 9：Claudication distance (CD) assessed by a a 6-minute walk test (6-MWT) for PAD patients treated by Nordic walking programs in comparison to control group.**

|  |  | **Nordic Walking program** | | | | | | **Control group** | | | | | |
| --- | --- | --- | --- | --- | --- | --- | --- | --- | --- | --- | --- | --- | --- |
| **ID** | **Study** | **Baseline**  **N** | **Post-intervention**  **N** | **Baseline** | **4-6weeks** | **12weeks** | **24weeks** | **Baseline**  **N** | **Post-intervention**  **N** | **Baseline** | **4-6weeks** | **12weeks** | **24weeks** |
| NO.1 | Spafford 2014 [26] | 28 | 19 | 178±77 | NR | 232±107 | NR | 24 | 19 | 130±71 | NR | 163±67 | NR |

NR=not reported. The control group of Spafford et al. [26] received standard home exercise therapy.

**Supplementary Table 10：exercise duration assessed by a treadmill test for PAD patients treated by Nordic walking programs in comparison to control group.**

|  |  | **Nordic Walking program** | | | | | | **Control group** | | | | | |
| --- | --- | --- | --- | --- | --- | --- | --- | --- | --- | --- | --- | --- | --- |
| **ID** | **Study** | **Baseline**  **N** | **Post-intervention**  **N** | **Baseline** | **4-6weeks** | **12weeks** | **24weeks** | **Baseline**  **N** | **Post-intervention**  **N** | **Baseline** | **4-6weeks** | **12weeks** | **24weeks** |
| NO.1 | Langbein 2002 [24] | 27 | NR | 10.43± 4.1 | 12.8± 4.87 | 14.62± 4.53 | 15.73±4.47 | 25 | NR | 11.07±4.7 | 10.03±4.1 | 10.07±3.73 | 10.33±4.67 |

NR=not reported. The control group of Langbein et al. [24] received non routine exercise therapy.
