# Supplementary material for: Effect of Nordic walking on walking ability in patients with peripheral arterial disease: a meta-analysis
Source: PLoS One. 2025 Mar 10;20(3):e0316092. doi: 10.1371/journal.pone.0316092 (PMC11892863; doi:10.1371/journal.pone.0316092)
Supplement: S1 File — (DOCX) [file pone.0316092.s003.docx]

**S2 Table.** Excluded studies and reason (N =153 )

| **No.** | **Author/year** | **Title** | **Reason(s) for exclusion** |
| --- | --- | --- | --- |
| 1 | Oakley et al., 2008 | Nordic poles immediately improve walking distance in patients with intermittent claudication | Repetitive literature（5 articles） |
| 2 | Collins et al., 2012 | Comparison of walking with poles and traditional walking for peripheral arterial disease rehabilitation | Repetitive literature（4 articles） |
| 3 | Tschentscher et al., 2013 | Health benefits of nordic walking: A systematic review | Repetitive literature（2 articles） |
| 4 | Spafford et al., 2014 | Randomized clinical trial comparing Nordic pole walking and a standard home exercise programme in patients with intermittent claudication | Repetitive literature（5 articles） |
| 5 | Gardner et al., 2015 | Exercise rehabilitation for peripheral artery disease: An exercise physiology perspective with special emphasis on the emerging trend of home-based exercise | Repetitive literature（2 articles） |
| 6 | Bulinska et al., 2016 | Nordic pole walking improves walking capacity in patients with intermittent claudication: a randomized controlled trial | Repetitive literature（6 articles） |
| 7 | Skórkowska-Telichowska et al., 2016 | Nordic walking in the second half of life | Repetitive literature（2 articles） |
| 8 | Cugusi et al., 2017 | Nordic walking for individuals with cardiovascular disease: A systematic review and meta-analysis of randomized controlled trials | Repetitive literature（4 articles） |
| 9 | Girold et al., 2017 | Nordic walking versus walking without poles for rehabilitation with cardiovascular disease: randomized controlled trial | Repetitive literature（6 articles） |
| 10 | Oakley et al., 2017 | A Three Month Home Exercise Programme Augmented with Nordic Poles for Patients with Intermittent Claudication Enhances Quality of Life and Continues to Improve Walking Distance and Compliance After One Year | Repetitive literature（8 articles） |
| 11 | Pasiak et al., 2017 | Applied methods of exercise based therapy for the extension of walking distance in patients with intermittent claudication | Repetitive literature（2 articles） |
| 12 | Unknown et al., 2018 | One year on: Test your knowledge from the previous year | Repetitive literature（2 articles） |
| 13 | Golledge et al., 2008 | Systematic Review and Meta-analysis of Clinical Trials Examining the Benefit of Exercise Programmes Using Nordic Walking in Patients With Peripheral Artery Disease | Repetitive literature（5 articles） |
| 14 | Kropielnicka et al., 2018 | Influence of the physical training on muscle function and walking distance in symptomatic peripheral arterial disease in elderly | Repetitive literature（5 articles） |
| 15 | Nordanstig et al., 2018 | Is There a Claudicating Elephant in the Room? | Repetitive literature（2 articles） |
| 16 | Golledge et al., 2019 | Response to ‘Re. Systematic Review and Meta-analysis of Clinical Trials Examining the Benefit of Exercise Programs Using Nordic Walking in Patients with Peripheral Artery Disease’ | Repetitive literature（5 articles） |
| 17 | Rosloniec et al., 2019 | DOES USE OF NORDIC POLES IMPROVE WALKING DISTANCE IN PATIENTS WITH INTERMITTENT CLAUDICATION? | Repetitive literature（2 articles） |
| 18 | Spannbauer et al., 2019 | Intermittent Claudication in Physiotherapists' Practice | Repetitive literature（4 articles） |
| 19 | Wee et al., 2019 | Nordic Walking in Patients with Peripheral Artery Disease | Repetitive literature（4 articles） |
| 20 | Dziubek et al., 2020 | Effects of Physical Rehabilitation on Spatiotemporal Gait Parameters and Ground Reaction Forces of Patients with Intermittent Claudication | Repetitive literature（5 articles） |
| 21 | Jansen et al., 2020 | Modes of exercise training for intermittent claudication | Repetitive literature（3 articles） |
| 22 | Lanzi et al., 2021 | Gait changes after supervised exercise training in patients with symptomatic lower extremity peripheral artery disease | Repetitive literature（4 articles） |
| 23 | Lanzi et al., 2021 | Improvement in 6-minute walking distance after supervised exercise training is related to changes in quality of life in patients with lower extremity peripheral artery disease | Repetitive literature（2 articles） |
| 24 | Mesa-Vieira et al., 2021 | Psychosocial risk factors in cardiac rehabilitation: Time to screen beyond anxiety and depression | Repetitive literature（2 articles） |
| 25 | Ney et al., 2021 | Multimodal supervised exercise training is effective in improving long term walking performance in patients with symptomatic lower extremity peripheral artery disease | Repetitive literature（2 articles） |
| 26 | Qi et al., 2022 | Exercise for peripheral artery disease | Repetitive literature（2 articles） |
| 27 | Ibeggazene et al., 2023 | A systematic review of exercise intervention reporting quality and dose in studies of intermittent claudication | Repetitive literature（2 articles） |
| 28 | Marzolini et al., 2023 | Determining the Optimal Type of Exercise for People with Symptomatic Lower Extremity Peripheral Artery Disease: Is There a One-Size-Fits-All Approach? | Repetitive literature（2 articles） |
| 29 | Sandberg et al., 2023 | Impact of walk advice alone or in combination with supervised or home-based structured exercise on patient-reported physical function and generic and disease-specific health related quality of life in patients with intermittent claudication, a secondary analysis in a randomized clinical trial | Repetitive literature（6 articles） |
| 30 | Sandberg et al., 2023 | Effectiveness of supervised exercise, home-based exercise, or walk advice strategies on walking performance and muscle endurance in patients with intermittent claudication (SUNFIT trial): a randomized clinical trial | Repetitive literature（6 articles） |
| 31 | Sandberg et al., 2023 | The Impact of Nordic Pole Walk Advice Alone or in Combination With Exercise Strategies on Daily Physical Activity in Patients With Intermittent Claudication: A Randomized Clinical Trial | Repetitive literature（2 articles） |
| 32 | Tremblay et al., 2023 | The Effect of Exercise Modalities on Walking Capacity in Patients with Intermittent Claudication: A NETWORK META-ANALYSIS | Repetitive literature（3 articles） |
| 33 | Ulfsdottir et al, 2023 | Cost-Effectiveness of Exercise Therapy in Patients with Intermittent Claudication—A Comparison of Supervised Exercise, Home-Based Structured Exercise, and Walk Advice from the SUNFIT Trial | Repetitive literature（5 articles） |
| 34 | Kambič et al, 2024 | Resistance Training in Cardiac Rehabilitation PAST, PRESENT, AND FUTURE | Repetitive literature（2 articles） |
|  |  |  | There are a total of 123 repeated references |
| 1 | Anonymous et al, 2008 | Peripheral Arterial Disease - Diagnosis and Treatment: A Systematic Review | Review |
| 2 | Cugusi, et al., 2017 | Nordic walking for individuals with cardiovascular disease: A systematic review and meta-analysis of randomized controlled trials | Review |
| 3 | Gardner et al, 2015 | Exercise rehabilitation for peripheral artery disease: An exercise physiology perspective with special emphasis on the emerging trend of home-based exercise | Review |
| 4 | Golledge et al, 2018 | Systematic Review and Meta-analysis of Clinical Trials Examining the Benefit of Exercise Programmes Using Nordic Walking in Patients With Peripheral Artery Disease | Review |
| 5 | Hap et al, 2021 | Patients with Diabetes Complicated by Peripheral Artery Disease: The Current State of Knowledge on Physiotherapy Interventions | Review |
| 6 | Anonymous et al, 2023 | A systematic review of exercise intervention reporting quality and dose in studies of intermittent claudication | Review |
| 7 | Jansen et al, 2020 | Modes of exercise training for intermittent claudication | Review |
| 8 | Kawamoto et al, 2014 | Effect of weight loss on central systolic blood pressure in elderly community-dwelling persons | Review |
| 9 | Morgulec-Adamowicz et al, 2011 | Nordic walking - A new form of adapted physical activity (a literature review) | Review |
| 10 | Skórkowska-Telichowska et al, 2016 | Nordic walking in the second half of life | Review |
| 11 | Tremblay et al, 2023 | The Effect of Exercise Modalities on Walking Capacity in Patients With Intermittent Claudication: A NETWORK META-ANALYSIS | Review |
| 12 | Tschentscher et al, 2013 | Health benefits of nordic walking: A systematic review | Review |
| 13 | Fiodorenko-Dumas et al., 2017 | Physical activity - related changes in ADMA and vWF levels in patients with type 2 diabetes: A preliminary study | Not PAD or IC |
| 14 | Lejczak., 2011 | 12-week Nordic Walking training improves physical fitness and quality of life in patients with systolic heart failure | Not PAD or IC |
| 15 | Lejczak., 2011 | Effects of 12-week Nordic Walking training on physical fitness and quality oflife in patients with chronic systolic heart failure | Not PAD or IC |
| 16 | Net., 2013 | The Effects of Omega-3 Fatty Acids on Peripheral Arterial Disease II | Not NW |
| 17 | Omarjee., 2019 | Effects of sildenafil on maximum walking time in patients with arterial claudication: the ARTERIOFIL study | Not NW |
| 18 | Collins., 2003 | PoleStriding exercise and vitamin E for management of peripheral vascular disease | The NW group combined medication |
| 19 | Sandberg., 2023 | The Impact of Nordic Pole Walk Advice Alone or in Combination With Exercise Strategies on Daily Physical Activity in Patients With Intermittent Claudication: a Randomized Clinical Trial | The control group also contained NW |
| 20 | Sandberg., 2022 | The impact of walk advice and exercise strategies on daily physical activity in patients with intermittent claudication - A randomized controlled trial | The control group also contained NW |
| 21 | Fritschi.，2012 | Workshop | Conference Abstract |
| 22 | Spafford., 2014 | Nordic Pole Walking is more effective than a standard Home Exercise Programme in improving walking distance in patients with intermittent claudication: A prospective randomised study | Conference Abstract |
| 23 | Rosloniec., 2019 | DOES USE OF NORDIC POLES IMPROVE WALKING DISTANCE IN PATIENTS WITH INTERMITTENT CLAUDICATION? | Conference Abstract |
| 24 | Rosloniec., 2016 | EFFECTS OF NORDIC POLE WALKING ON OXIDATIVE STRESS AND WALKING ABILITIES IN PATIENTS WITH INTERMITTENT CLAUDICATION | Conference Abstract |
| 25 | Rosloniec., 2018 | THE EFFECTS OF NORDIC WALKING TRAINING ON BLOOD ANTIOXIDANT DEFENCE IN PATIENTS WITH INTERMITTENT CLAUDICATION | Conference Abstract |
| 26 | Lejczak.，2011 | 12-week Nordic Walking training improves physical fitness and quality of life in patients with systolic heart failure | Conference Abstract |
| 27 | Saggar., 2022 | POST HOC ANALYSIS OF CLINICAL CHARACTERISTICS AND OUTCOMES IN PATIENTS WITH IDIOPATHIC PULMONARY FIBROSIS (IPF) BY PULMONARY VASCULAR PHENOTYPE FROM CAPACITY | Conference proceeding |
| 28 | Cugusi., 2017 | Nordic walking and cardiovascular disease prevention: A systematic review of randomized controlled trials | Conference Abstract |
| 29 | Ulfsdottir.,2023 | Cost-Effectiveness of Exercise Therapy in Patients with Intermittent Claudication-A Comparison of Supervised Exercise, Home-Based Structured Exercise, and Walk Advice from the SUNFIT Trial | Not meeting the outcome criteria |
| 30 | Peng et al, 2023 | The study of functional electrical stimulation on motor functional recovery and the expression of gfap and syn in the penumbra around the cerebral infarction of rats | Dissertation/Thesis |
