# Supplementary material for: Effect of Nordic walking on walking ability in patients with peripheral arterial disease: a meta-analysis
Source: PLoS One. 2025 Mar 10;20(3):e0316092. doi: 10.1371/journal.pone.0316092 (PMC11892863; doi:10.1371/journal.pone.0316092)
Supplement: S2 File — (DOCX) [file pone.0316092.s004.docx]

**S3 Table.** Quality assessment of randomized controlled trials and pseudo-random controlled trials using Cochrane risk of bias tool.

| Study | Design | Random sequence generation（selection bias） | Allocation concealment（selection bias） | Blinding of participants and personnel（performance bias） | Blinding of outcome assessment（detection bias） | Incomplete outcome data（attrition bias） | Selective reporting （reporting bias） | Other bias | Quality |
| --- | --- | --- | --- | --- | --- | --- | --- | --- | --- |
| Collins 2012 [23] | RCT | Yes | Yes | NO | Unclear | Yes | Yes | Yes | Low |
| Langbein 2002 [24] | RCT | Yes | Yes | NO | Unclear | Yes | Yes | Yes | Low |
| Collins（duration）2012 [25] | RCT | Yes | Yes | NO | Unclear | Yes | Yes | Yes | Low |
| Spafford 2014 [26] | RCT | Yes | Yes | NO | Unclear | NO | Yes | Yes | moderate |
| Girold 2017 [12] | RCT | Yes | Yes | NO | Yes | Yes | Yes | Yes | Low |
| Bulinska 2016 [29] | PRCT | NO | NO | NO | Unclear | NO | Yes | NO | High |
| Kropielnicka 2018 [27] | PRCT | NO | NO | NO | Unclear | NO | Yes | Yes | High |
| Dziubek 2020 [28] | PRCT | NO | NO | NO | Unclear | NO | Yes | Yes | High |

Those meeting 5 or more criteria were judged to carry a low risk of bias, those fulfilling 3–4 criteria were assigned a medium risk of bias, and those satisfying fewer than 3 criteria

were labeled as high risk. RCT：randomized controlled trials；PRCT：pseudo-random controlled trials.
