# Supplementary material for: Effect of Nordic walking on walking ability in patients with peripheral arterial disease: a meta-analysis
Source: PLoS One. 2025 Mar 10;20(3):e0316092. doi: 10.1371/journal.pone.0316092 (PMC11892863; doi:10.1371/journal.pone.0316092)
Supplement: S4 File — (DOCX) [file pone.0316092.s006.docx]

**S7 Table.** PICOS principle

| **PICOS principle** | |
| --- | --- |
| **Patient/Population:** | Peripheral arterial disease and patients with intermittent claudication. |
| **Intervention:** | Nordic Walking. |
| **Comparison：** | Supervised exercise therapy, standard home exercise program, and non-exercise routine medical group. |
| **Outcomes:** | Primary outcomes:maximum walking distance (MWD), claudication distance (CD), and exercise duration. |
| **Study design:** | Randomized controlled trials (RCT) and pseudo-randomised controlled trials (PRCT) . |
