# Supplementary material for: Effect of Nordic walking on walking ability in patients with peripheral arterial disease: a meta-analysis
Source: PLoS One. 2025 Mar 10;20(3):e0316092. doi: 10.1371/journal.pone.0316092 (PMC11892863; doi:10.1371/journal.pone.0316092)
Supplement: S5 File — (DOCX) [file pone.0316092.s007.docx]

**S8 Table.** The search and screening strategy

| Electronic data | Results | search strategy (search performed on 9 April 2024) |
| --- | --- | --- |
| PubMed | 7 | ((("Peripheral Arterial Disease"[Mesh]) OR ((((((((((((((Arterial Disease, Peripheral[Title/Abstract]) OR (Arterial Diseases, Peripheral[Title/Abstract])) OR (Disease, Peripheral Arterial[Title/Abstract])) OR (Diseases, Peripheral Arterial[Title/Abstract])) OR (Peripheral Arterial Diseases[Title/Abstract])) OR (Peripheral Artery Disease[Title/Abstract])) OR (Artery Disease, Peripheral[Title/Abstract])) OR (Artery Diseases, Peripheral[Title/Abstract])) OR (Disease, Peripheral Artery[Title/Abstract])) OR (Diseases, Peripheral Artery[Title/Abstract])) OR (Peripheral Artery Diseases[Title/Abstract])) OR (peripheral arterial occlusive disease[Title/Abstract])) OR (peripheral vascular disease[Title/Abstract])) OR (peripheral artery disease[Title/Abstract]))) OR (("Intermittent Claudication"[Mesh]) OR ((((Claudication, Intermittent[Title/Abstract]) OR (Intermittent limp[Title/Abstract])) OR (limb ischaemia[Title/Abstract])) OR (limb ischemia[Title/Abstract])))) AND (("Nordic Walking"[Mesh]) OR (((((Walking, Nordic[Title/Abstract]) OR (Pole Walking[Title/Abstract])) OR (Walking, Pole[Title/Abstract])) OR (pole walking[Title/Abstract])) OR (pole striding[Title/Abstract]))) |
| Embase | 37 | #1 'intermittent claudication'/exp OR 'intermittent claudication'  #2 'claudication, intermittent':ab,ti OR 'intermittent limp':ab,ti  #3 #1 OR #2  #4 'peripheral arterial disease'/exp OR 'peripheral arterial disease'  #5 'arterial disease, peripheral':ab,ti OR 'arterial diseases, peripheral':ab,ti OR 'disease, peripheral arterial':ab,ti OR 'diseases, peripheral arterial':ab,ti OR 'peripheral arterial diseases':ab,ti OR 'peripheral artery disease':ab,ti OR 'artery disease, peripheral':ab,ti OR 'artery diseases, peripheral':ab,ti OR 'disease, peripheral artery':ab,ti OR 'diseases, peripheral artery':ab,ti OR 'peripheral artery diseases':ab,ti  #6 #4 OR #5  #7 #3 OR #6  #8 'nordic walking'/exp OR 'nordic walking'  #9 'walking, nordic':ab,ti OR 'pole walking':ab,ti OR 'walking, pole':ab,ti OR 'walk advice':ab,ti  #10 #8 OR #9  #11 'randomized controlled trial':ab,ti OR 'randomized':ab,ti OR 'placebo':ab,ti  #12 #7 AND #10 AND #11 |
| Web of Science | 27 | #1: (TI=(Peripheral Artery Disease)) OR AB=(Peripheral Artery Disease) and Preprint Citation Index (Exclude – Database)  #2: (((TI=(Peripheral Artery Disease)) OR AB=(Peripheral Artery Disease)) AND TI=(Arterial Disease, Peripheral OR Arterial Diseases, Peripheral OR Disease, Peripheral Arterial OR Diseases, Peripheral Arterial OR Peripheral Arterial Diseases OR Peripheral Artery Disease OR Artery Disease, Peripheral OR Artery Diseases, Peripheral OR Disease, Peripheral Artery OR Diseases, Peripheral Artery OR Peripheral Artery Diseases OR peripheral arterial occlusive disease OR peripheral vascular disease OR peripheral artery disease )) OR AB=(Arterial Disease, Peripheral OR Arterial Diseases, Peripheral OR Disease, Peripheral Arterial OR Diseases, Peripheral Arterial OR Peripheral Arterial Diseases OR Peripheral Artery Disease OR Artery Disease, Peripheral OR Artery Diseases, Peripheral OR Disease, Peripheral Artery OR Diseases, Peripheral Artery OR Peripheral Artery Diseases OR peripheral arterial occlusive disease OR peripheral vascular disease OR peripheral artery disease ) and Preprint Citation Index (Exclude – Database)  #3: #2 OR #1 and Preprint Citation Index (Exclude – Database)  #4: (TI=(Intermittent Claudication )) OR AB=(Intermittent Claudication ) and Preprint Citation Index (Exclude – Database)  #5: (((TI=(Intermittent Claudication )) OR AB=(Intermittent Claudication )) AND TI=( Claudication, Intermittent OR Intermittent limp OR limb ischaemia OR limb ischemia )) OR AB=( Claudication, Intermittent OR Intermittent limp OR limb ischaemia OR limb ischemia ) and Preprint Citation Index (Exclude – Database)  #6: #4 OR #5 and Preprint Citation Index (Exclude – Database)  #7: #3 OR #6 and Preprint Citation Index (Exclude – Database)  #8: (TI=(Nordic Walking)) OR AB=(Nordic Walking) and Preprint Citation Index (Exclude – Database)  #9: (((TI=(Nordic Walking)) OR AB=(Nordic Walking)) AND TI=(Walking, Nordic OR Pole Walking OR Walking, Pole OR pole walking OR pole striding )) OR AB=(Walking, Nordic OR Pole Walking OR Walking, Pole OR pole walking OR pole striding ) and Preprint Citation Index (Exclude – Database)  #10: #8 OR #9 and Preprint Citation Index (Exclude – Database)  #11: #10 AND #7 and Preprint Citation Index (Exclude – Database) |
| The Cochrane Library | 27 | #1 MeSH descriptor: [Peripheral Arterial Disease] explode all trees  #2 Arterial Disease,Peripheral OR Arterial Diseases,PeripheralOR Disease, Peripheral Arterial OR Diseases, Peripheral Arterial OR Peripheral Arterial Diseases OR Peripheral Artery Disease OR Artery Disease, Peripheral OR Artery Diseases,Peripheral OR Disease, Peripheral Artery OR Diseases, Peripheral Artery OR Peripheral Artery Diseases OR peripheral arterial occlusive disease ORperipheral vascular disease OR peripheral artery disease  #3 #1 OR #2  #4 MeSH descriptor: [Intermittent Claudication] explode all trees  #5 Claudication,Intermittent OR Intermittent limp OR limb ischaemia ORlimb ischemia  #6 #4 OR #5  #7 #3 OR #6  #8 MeSH descriptor: [Nordic Walking] explode all trees  #9 Walking, Nordic OR Pole Walking OR Walking, Pole OR pole walking OR pole striding  #10 #8 OR #9  #11 #7 AND #10 |
| EBSCO host | 7 | S1 SU Peripheral Artery Disease OR SU (Arterial Disease，Peripheral OR Arterial Diseases,Peripheral OR Disease,Peripheral Arterial OR Diseases, Peripheral Arterial OR Peripheral Arterial Diseases OR Peripheral Artery Disease OR Artery Disease, Peripheral OR Artery Diseases, Peripheral OR Disease, Peripheral Artery OR Diseases, Peripheral Artery OR Peripheral Artery Diseases OR peripheral arterial occlusive disease OR peripheral vascular disease OR peripheral artery disease )  S2 SU Intermittent Claudication OR SU (Claudication, Intermittent OR Intermittent limp OR limb Ischaemia OR limb ischemia )  S3 (SU Intermittent Claudication OR SU Claudication, Intermittent OR Intermittent limp OR limb ischaemia OR limb ischemia) OR(S1)  S4 SU Nordic Walking OR SU( Walking, Nordic OR Pole Walking OR Walking, Pole OR pole walking OR pole striding )  S5 (SU Nordic Walking OR SU Walking, Nordic OR Pole Walking OR Walking, Pole OR pole walking OR pole striding) AND(S3 AND S4) |
| Ovid | 19 | #1 Peripheral Arterial Disease/  #2 (Arterial Disease, Peripheral or Arterial Diseases, Peripheral or Disease, Peripheral Arterial or Diseases,  Peripheral Arterial or Peripheral Arterial Diseases or Peripheral Artery Disease or Artery Disease, Peripheral or2 Artery Diseases, Peripheral or Disease, Peripheral Artery or Diseases, Peripheral Artery or Peripheral Artery  Diseases or peripheral arterial occlusive disease or peripheral vascular disease or peripheral artery disease).ab,ti,kw.  #3 Intermittent Claudication/  #4 (Claudication, Intermittent or Intermittent limp or limb ischaemia or limb ischemia).ab,ti,kw.  # 5 1 or 2 or 3 or 4  #6 Nordic Walking/  #7 (Walking, Nordic or Pole Walking or Walking, Pole or pole striding or pole walking).ab,ti,kw.  #8 6 or 7  #9 5 and 8 |
| Scopus | 36 | #1 TITLE-ABS-KEY ( "Arterial Disease, Peripheral" OR "Arterial Diseases, Peripheral" OR "Disease, Peripheral Arterial" OR "Diseases, Peripheral Arterial" OR "Peripheral Arterial Diseases " OR "Artery Disease, Peripheral" OR "Peripheral Artery Disease" OR "Artery Diseases, Peripheral" OR "Disease, Peripheral Artery" OR "Diseases, Peripheral Artery" OR "Peripheral Artery Diseases" OR " peripheral vascular disease" OR "peripheral arterial occlusive disease " OR "peripheral artery disease" OR "Peripheral Artery Disease" )  #2 TITLE-ABS-KEY ( "Claudication, Intermittent " OR "Intermittent limp" OR "limb ischaemia" OR " limb ischemia ." OR "Intermittent Claudication " )  #3 ( TITLE-ABS-KEY ( "Arterial Disease, Peripheral" OR "Arterial Diseases, Peripheral" OR "Disease, Peripheral Arterial" OR "Diseases, Peripheral Arterial" OR "Peripheral Arterial Diseases " OR "Artery Disease, Peripheral" OR "Peripheral Artery Disease" OR "Artery Diseases, Peripheral" OR "Disease, Peripheral Artery" OR "Diseases, Peripheral Artery" OR "Peripheral Artery Diseases" OR " peripheral vascular disease" OR "peripheral arterial occlusive disease " OR "peripheral artery disease" OR "Peripheral Artery Disease" ) ) OR ( TITLE-ABS-KEY ( "Claudication, Intermittent " OR "Intermittent limp" OR "limb ischaemia" OR " limb ischemia ." OR "Intermittent Claudication " ) )  #4 TITLE-ABS-KEY ( "Walking, Nordic" OR "Pole Walking" OR "Walking, Pole" OR "pole walking" OR "pole striding" OR "Nordic Walking" )  #5 ( ( TITLE-ABS-KEY ( "Arterial Disease, Peripheral" OR "Arterial Diseases, Peripheral" OR "Disease, Peripheral Arterial" OR "Diseases, Peripheral Arterial" OR "Peripheral Arterial Diseases " OR "Artery Disease, Peripheral" OR "Peripheral Artery Disease" OR "Artery Diseases, Peripheral" OR "Disease, Peripheral Artery" OR "Diseases, Peripheral Artery" OR "Peripheral Artery Diseases" OR " peripheral vascular disease" OR "peripheral arterial occlusive disease " OR "peripheral artery disease" OR "Peripheral Artery Disease" ) ) OR ( TITLE-ABS-KEY ( "Claudication, Intermittent " OR "Intermittent limp" OR "limb ischaemia" OR " limb ischemia." OR "Intermittent Claudication " ) ) ) AND ( TITLE-ABS-KEY ( "Walking, Nordic" OR "Pole Walking" OR "Walking, Pole" OR "pole walking" OR "pole striding" OR "Nordic Walking" ) )  #6 ( ( TITLE-ABS-KEY ( "Arterial Disease, Peripheral" OR "Arterial Diseases, Peripheral" OR "Disease, Peripheral Arterial" OR "Diseases, Peripheral Arterial" OR "Peripheral Arterial Diseases " OR "Artery Disease, Peripheral" OR "Peripheral Artery Disease" OR "Artery Diseases, Peripheral" OR "Disease, Peripheral Artery" OR "Diseases, Peripheral Artery" OR "Peripheral Artery Diseases" OR " peripheral vascular disease" OR "peripheral arterial occlusive disease " OR "peripheral artery disease" OR "Peripheral Artery Disease" ) ) OR ( TITLE-ABS-KEY ( "Claudication, Intermittent " OR "Intermittent limp" OR "limb ischaemia" OR " limb ischemia." OR "Intermittent Claudication " ) ) ) AND ( TITLE-ABS-KEY ( "Walking, Nordic" OR "Pole Walking" OR "Walking, Pole" OR "pole walking" OR "pole striding" OR "Nordic Walking" ) ) |
