# Supplementary material for: Effect of Nordic walking on walking ability in patients with peripheral arterial disease: a meta-analysis
Source: PLoS One. 2025 Mar 10;20(3):e0316092. doi: 10.1371/journal.pone.0316092 (PMC11892863; doi:10.1371/journal.pone.0316092)
Supplement: Attachment — Submitted filename: Rebuttal letter.docx [file pone.0316092.s009.docx]

Rebuttal letter

Dear Editor and Reviewer，

Firstly, I sincerely thank the editors and reviewers for taking the time and effort to review my manuscript, which has given me the opportunity to improve its quality. I am very happy about this. The reviewer provided a lot of valuable suggestions in the view letter, and I carefully read and made revisions to each suggestion. During this process, I learned a lot of knowledge and gained a deeper understanding of the exercise method of Nordic walking. All the parts in the manuscript that have been modified according to the suggestions of the reviewer have been marked in red. Now, I will respond to every point raised by the editor and reviewers.

**Editor’s comments:**

1. Thank you very much for letting me know which content needs to be submitted. I will submit a rebuttal letter, revise the manuscript, and modify the unmarked manuscript according to your requirements.
2. I have revised the overall format according to the editor's requirements, checked for errors in the manuscript, and added the DOI numbers of the references.
3. I have registered on the ORCID iD of the corresponding author. Thank you for letting me know.
4. We have verified the fund number and will make the necessary changes during submission. Thank you for the editor's reminder.
5. We have removed the figures from the manuscript as requested by the editor and will upload them as a separate file, while retaining the tables in the manuscript.
6. We have checked the funding number and it is correct. This study was supported by a grant from the Jinhua Science and Technology Research Program Project of Public Welfare Category in 2021 (2021-4-228).

**Reviewer’s comments:**

**Comment:**

1. **a.** I am very grateful that you pointed out the incorrect part of my expression. I have now made revisions to lines 17-23. The result is located on lines 18-21 of page 1 of the revised manuscript: “**compared with supervised exercise therapy (SET), NW was not associated with an increase in maximum walking distance (MWD) and claudication distance (CD) in PAD patients, whether during treadmill tests or 6-minute walk tests (6-MWT), and the results were not statistically significant.**”

**b.** I am very grateful that you pointed out the incorrect part of my expression. Now, following your suggestion, I have made modifications to lines 434-439 by removing the SMD, Z, and p values from the discussion section. The result is located on page 21, lines 352-353 of the revised manuscript: “**compared with SET, NW was not associated with an increase in MWD and CD in PAD patients, whether during treadmill tests or 6-MWT.**”

**2.** I am very grateful for the question you raised. Because Figure 11 is based on only one experiment, I have removed it from the manuscript based on your suggestion. In Figure 9, the mean difference between the Nordic group and the control group is negative, equal to -0.41, indicating that the mean and standard deviation of the Nordic group are smaller than those of the control group. Therefore, patients in the control group have longer exercise time and persist for a longer period of time. The same applies to Figure 10, which is consistent with the actual results. I apologize for the confusion caused to the reviewer due to the incorrect direction of the names marked on the left and right sides below the forest map. I have now made revisions to Figures 9 and 10.

**3.** I am very grateful for the question you raised. Sorry, due to the limited literature included, it cannot reflect the impact of unsupervised exercise compared to non exercise on the walking ability of PAD patients. In this section, I cited other scholars' research, which can be found on pages 23-24, lines 405-419: “**For many PAD patients, HET with convenient exercise methods and low prices are easier for them to accept [35], and have significant advantages compared to non exercise conventional treatments. In a randomized clinical trial [37] conducted by McDermott et al. in 2021, participants with PAD were randomly divided into three groups: low-intensity walking exercise (n=116), high-intensity walking exercise (n=124), and non exercise control group (n=65). Both exercise groups were required to conduct walking exercises in an unsupervised environment. The results showed that the effect of low-intensity family exercise was significantly lower than that of high-intensity family exercise, and there was no significant difference in improving 6-minute walking distance (6MWD) compared to non exercise control. The 6MWD of the high-intensity exercise group increased from 338.1 m to 371.2 m [within-group mean change = 34.5 m, 95%Cl (20.1, 48.9 m), P<0.001]; non exercise control group increased from 328.1 m at baseline to 317.5 m at the 12-month follow-up [within-group mean change = -15.1 m, 95%Cl (-35.8, 5.7 m), P = 0.10]**.”

**4.** I greatly appreciate your suggestion and have now removed Figure 11 from the manuscript.

**5.** I greatly appreciate your suggestions for revisions. I have removed lines 392-397 from the manuscript and made changes to lines 449-452 under discussion. The results are located on page 21, lines 356-357:“**Since there is only one research result of CD and exercise duration included in the literature, no meta-analysis was conducted.**”

**6.** I greatly appreciate your suggestions for revision. I have carefully read your feedback and made the necessary changes. I have removed the column for supervision from Table 1 and placed the word 'supervision' before NW. **Please refer to Figure 1 for the results.** And I also think your feedback on my discussion section was very good, which taught me a lot. The results are located on pages 21-23, lines 360-387：“**This meta-analysis included two unsupervised trials in the control group. Langbein et al. [24] used supervised NW, while Spafford et al. [26] used unsupervised NW. Compared with the control group, they were more effective in increasing MWD and improving walking function in PAD patients. However, it is worth noting that from the data of two studies from intervention to week 12, supervised NW was significantly better than unsupervised NW in increasing patient MWD (1001 ± 913m vs 652 ± 373m). This may mean that the extent to which NW intervention increases MWD in patients compared to unsupervised therapy is influenced by the supervision itself, rather than the exercise style. In 2007, Wind et al. conducted a systematic review comparing SET with unsupervised exercise therapy (exercise guidance) and found that SET can increase the pain-free walking distance (PFWD) and MWD of patients with intermittent claudication [31]. The results were more supportive of SET [WMD = 143.81, 95%Cl (5.81281.81), Z=2.04, P=0.04; WMD = 250.40, 95%Cl（192.35，308.45，Z=8.45，P＜0.00001], But whether it is more effective than exercise guidance requires further research. Another meta-analysis [32] published by Fokkenrood et al. in Cochrane library in 2013 also showed that after three months of intervention, participants who participated in supervised walking exercise walked 180 meters more than those who participated in unsupervised walking exercise, which improved their walking ability (MWD and PFWD) to a greater extent [SMD = 0.69, 95% Cl (0.51, 0.86), Z=7.80, P<0.00001; SMD = 0.70，95%Cl (0.52, 0.89), Z=7.37，P＜0.00001]. In the same year, Parmenter et al. [33] compared supervised whole body high-intensity PRT (H-PRT) with routine treatment of unsupervised walking for 6 months and found that H-PRT significantly improved the 6-minute walking distance (6MWD) of elderly people with intermittent claudication symptoms, while unsupervised walking exercise did not [n=8; MD = 62.6 ± 58.0 m, P=0.02; n=7; MD = -9.9 ± 52.9 m]. The above studies all show that the supervised exercise group has greater statistical benefits compared to the unsupervised exercise group.**”In addition, I have also made revisions to other parts of the discussion and checked for some errors, but the changes will not affect the content and framework of the manuscript. Due to word count limitations, they have been highlighted in red in the manuscript.

**7.** Thank you very much for your question. Based on your inquiry, my response is as follows: The results are based on week 12, as not all trials only intervened until week 12, some intervened until week 4, and some intervened until week 24. In order to avoid a large time span in the 24 week intervention trial, which also used data from week 12 for meta-analysis, I have revised this section in the manuscript, located on page 10, lines 195-204：“**By combining the effect sizes of MWD, CD, and exercise duration using data reported by the experimental and control groups at week 12. If there is no data available at the time of data extraction or the intervention period exceeds week 12, the most recent available time will be used. For example, if the Girold trial [12] only intervenes until week 4, the data at that time point will be used. The intervention period of Collins [23] and Langbein [24] was 24 weeks, and 5 studies [25-29] included in the trial were intervened until 12 weeks. In order to prevent the study time span from being too large, Collins [23] and Langbein's [24] study also used data from the 12th week for meta-analysis.**”

**8.** Thank you very much for identifying the errors in my manuscript. I have checked Table 1 and found that the sample size does include unanalyzed groups, which have been corrected. I apologize for my negligence.

**9.** Thank you very much for checking the errors in my manuscript. I have changed all the names in the manuscript to the author's last name.

**10.** I have already signed in the Data Extraction and Quality Assessment section, and the results are located on pages 8-9, lines 167 and 177：“**(Z.R.S. and J.Z.)**” I feel very impressed with the reviewer.

**Minor comments:**

A. Thank you very much for the editor's suggestion. We have rewritten lines 85-87 of the original manuscript. The result is located on page 4, lines 84-86:“**Since various RCTs have been implemented, it is more accurate to report whether separate RCTs generated conflicting evidence, where a meta-analysis can be helpful in settling such controversies.**”
B. Thank you very much for the editor's suggestion. Four years of meta-analysis have been added to the manuscript. The results are located on lines 88, 92, 97, and 111.
C. Thank you very much for the editor's suggestion. We have rewritten lines 94-97 of the original manuscript, and the result is located on page 5, lines 91-97: “**In 2018, Golledge et al. [18] aimed to explore the advantages of NW over other treatment methods in improving walking ability in patients with PAD. This study included five trials with different control group designs, including three SETs, one HET, and one routine treatment without exercise plan. The results showed that NW did not demonstrate superiority over other treatment methods in improving the walking ability of PAD patients.**”
D. I am very grateful that you have identified the grammar errors in my manuscript and have made the necessary revisions as suggested by the reviewer. The results are located on lines 71 and 81 respectively, and the word 'Second' has been removed from the text, resulting in line 97.
E. Thank you very much for pointing out the issue. I have revised all the results of the meta-analysis in the article. For example, rewriting the original lines 98-101 results in lines 97-104 on page 5:“**In 2020, Jansen et al. [19] published a meta-analysis in the authoritative Cochrane Library, which included three clinical trials of Nordic walking. The results showed that there was no statistically significant difference in maximum walking distance (MWD), pain-free walking distance (PFWD), and the Walking Impairment Questionnaire (WIQ) between the NW group and the control group [SMD = 0.04, 95% CI (-0.47 ，0.54), P=0.88; SMD = 0.21, 95% CI (-0.10 ，0.52), P=0.19; SMD = -2.30, 95% CI (-16.26 ，11.66), P=0.75], which was similar to the previous research results of Golledge [18].** ”
F. Thank you very much for your suggestion. I have added the importance of Cochrane in the meta-analysis section to the manuscript, and the results are located on page 5, lines 104-110: “**It is worth noting that the Systematic Reviews published in the Cochrane Library follow strict and unified work manual guidelines, have standardized format and content requirements, and maintain regular updates and improved feedback mechanisms. These characteristics are common. It ensures the high quality and authority of its output, usually exceeding the standards of non Cochrane systematic reviews. Therefore, Jansen et al.'s [19] research findings have high reference value in the academic community.** ”
G. Thank you very much for raising questions about my manuscript. You are correct. References 17 and 18 both focus on the impact of NW on PAD walking, but some of these trials not only investigated NW but also other cardiovascular diseases. Therefore, I only know of one trial that is entirely based on NW and PAD patients. I have also rewritten the manuscript, and the result is located on page 6, lines 115-125: “**In the comprehensive review of the four meta-analyses, only the intervention measures and research subjects of Golledge et al. [18] were fully focused on NW and PAD patients, with a literature search scope up to December 2017. This design enables direct and in-depth exploration of the impact of NW on PAD patients. The other three studies did not primarily analyze the benefits of NW for PAD patients. Cugusi et al.'s [17] study covered all randomized controlled trials prior to November 2016, adopting a broader inclusion criteria for the study population, including not only PAD patients but also other types of cardiovascular disease patients. Jansen [19] and Tremblay's [20] study focused on the population of PAD patients, but their focus was on evaluating the efficacy of different exercise methods in improving patients' walking ability**. ”
H. Thank you very much for your valuable suggestion. In the manuscript, I have rephrased sections 107-109 and added the years they cover. The results are located on lines 118 and 121 respectively, and I have provided a clearer explanation of why this study needs to conduct a meta-analysis again. The results are located on pages 6-7, lines 115-136: “**In the comprehensive review of the four meta-analyses, only the intervention measures and research subjects of Golledge et al. [18] were fully focused on NW and PAD patients, with a literature search scope up to December 2017. This design enables direct and in-depth exploration of the impact of NW on PAD patients. The other three studies did not primarily analyze the benefits of NW for PAD patients. Cugusi et al.'s [17] study covered all randomized controlled trials prior to November 2016, adopting a broader inclusion criteria for the study population, including not only PAD patients but also other types of cardiovascular disease patients. Jansen [19] and Tremblay's [20] study focused on the population of PAD patients, but their focus was on evaluating the efficacy of different exercise methods in improving patients' walking ability. Although they provide valuable reference information for selecting appropriate exercise plans for PAD patients in clinical practice by comparing different exercise intervention strategies. However, these four studies have limitations in the number of included literature, especially the 2023 meta-analysis [20] which only included one trial related to NW. In addition, Golledge et al. [18] analyzed walking ability based on the baseline number of participants and did not exclude participants who withdrew from the study midway. The outcome measure only involved the maximum walking distance (MWD), and did not directly explore the claudication distance (CD) as an outcome measure. Moreover, the study has been ongoing for seven years, during which other scholars may have published new research results, which may provide strong evidence for the comprehensive systematic evaluation of NW in this study**.”
I. Thank you very much for pointing out my mistake. I have filled in the missing words in the manuscript, and the result is located on page 10, line 196.
J. Thank you very much for your suggestion. I have added the sample size for each study baseline and at the end of the intervention in Table 1.
K. Thank you very much for your question. In Chinese, the meaning of an entry is to divide the content into items. For example, in Cochane quality assessment, it is divided into 7 entries, including "Random sequence generation", "Allocation concept", et al. In order to prevent misunderstanding of this word, I have rephrased this section and the result is located on page 17, lines 257-265: “**The results of the quality assessment are shown in Figure 3 and Figure 4. In the RCT, 3 articles reached low risk of bias and had high quality, while the remaining 2 articles had moderate bias; In Figure 2, if it meets the standard, it is marked as "+", and if it does not meet the standard, it is marked as "-". All included trials require approval from the local ethics committee in accordance with legal regulations. Participants are required to fill out informed consent forms before enrollment, record their height and weight, and teach how to use Nordic walking poles. Therefore, blinding cannot be used for both participants and assessors, and only one trial (Girold et al. [12]) reported blinding by the outcome assessors.** ”
L. Thank you very much for the reviewer's question. The limp trial refers to intermittent claudication, and the result is located on page 17, line 265: “**Many trials allowed 20% of subjects to drop out**”
M. Thank you very much for pointing out the issues raised by the reviewer. The phrase 'with Bulinska et al' has been removed from the manuscript. The modification result can be seen on page 17, line 268.

The above is my response to the comments raised by the editor and reviewer. Once again, I would like to express my sincere gratitude. The biggest gain during the submission process was the feedback on the manuscript due to insufficient content. Although there are still many shortcomings in my manuscript, I will work hard to make revisions and strive to meet your requirements. Thank you.

Looking forward to letters from editors and reviewers, wishing you a happy life!

Yours sincerely.

Zerong Sun, Yongdong Qian
