# Supplementary material for: Effect of Nordic walking on walking ability in patients with peripheral arterial disease: a meta-analysis
Source: PLoS One. 2025 Mar 10;20(3):e0316092. doi: 10.1371/journal.pone.0316092 (PMC11892863; doi:10.1371/journal.pone.0316092)
Supplement: Attachment — Submitted filename: Rebuttal_letter_auresp_2.docx [file pone.0316092.s010.docx]

Rebuttal letter

Dear Editor and Reviewer，

Firstly, I would like to sincerely thank the editor and reviewers for taking the time and effort to review my manuscript, which has given me the opportunity to improve its quality. I am very happy about this. The reviewer provided a lot of valuable suggestions in the view letter, and I carefully read and made revisions to each suggestion. During this process, I learned a lot of knowledge and gained a deeper understanding of the exercise method of Nordic walking. All the parts in the manuscript that have been modified according to the suggestions of the reviewer have been marked in red. Now, I will respond to every point raised by the editor and reviewers.

**Reviewer’s comments:**

1. I am very grateful for every suggestion made by the reviewer. In response to your questions about the figures, I have the following answers. **Figure 8** shows the meta-analysis results of supervised NW and SET on claudication distance in PAD patients under 6-MWT testing. This figure shows no statistical difference. The claudication distance refers to the distance that patients with peripheral arterial disease feel pain in their calves or thighs after starting to walk. The longer the claudication distance, the less likely the patient is to feel pain while walking, so a longer claudication distance is better. The effect size of Figure 8 is -0.09, and the standard deviation and mean of the NW group minus the mean and standard deviation of the control group are negative, indicating that claudication distance in the NW group is lower than that in the control group, and claudication distance in the control group is slightly longer. **Figure 9** shows the exercise duration of NW compared to SET. The longer the walking time, the better, with an effect size of -0.41, indicating that SET is superior to NW. Therefore, SET has a better exercise effect and prolongs the patient's walking time. This time, I changed the direction of the footnotes in **Figure 5-9** back, placing NW on the left and the control group on the right. I consulted some meta-analysis forest plot data and normally placed the intervention group on the left and the control group on the right. The larger the three outcome measures of meta-analysis, the better the patient's walking ability.
2. I fully agree with the reviewer's suggestion and have changed NW to supervised NW as per your request. The revised results can be found in lines **245, 254, 262, 271, 278, 285, 325, 327, 399, 400 and 403** of the manuscript.
3. I greatly appreciate the reviewer's suggestion and have revised the footnote for the control group in the figures to "Control".
4. I am very grateful to the reviewer for pointing out my grammar error. The revised result can be found in lines **227-229** of the manuscript.
5. I am very grateful to the reviewer for pointing out my grammar errors. The revised results are shown in lines **93-96** of the manuscript. The revised content is as follows: **Although they provide valuable reference information for selecting appropriate exercise plans for PAD patients in clinical practice by comparing different exercise intervention strategies, these four studies still have limitations in the number of included literature.**
6. I am very grateful for the valuable suggestions provided by the reviewer. I have streamlined the introduction according to the suggestions made by the reviewers. I have included **the advantages of Nordic walking in the fourth paragraph of the original introduction** in the discussion, which is now located in lines **404-414** of the manuscript. Similarly, regarding the four previously published meta-analyses, only the results section of the study was described, located in lines **77-92**. The excess content is placed in the first paragraph of the discussion, in lines **295-322** of the manuscript, and a brief explanation of what was added to the previous study is now located in lines **93-106** of the manuscript.
7. I am very grateful for the valuable suggestions provided by the reviewer. After thinking about it, I also believe that two different control groups should not be attributed to unsupervised exercise, as this description is inaccurate. Therefore, I have **removed the section comparing the results with unsupervised data and Figure 10**, and clearly stated in the Results, Discussion, and Limitations section that meta-analysis cannot be conducted, located at **240-244, 328-342, and 461-464** in the manuscript.
8. I am very grateful to the reviewer for pointing out my negligence. I have now explained in the text which two papers it is, and the results are located in lines **337-340** of the manuscript. The revised content is as follows: **It is worth noting that two studies [24] [26] from intervention to week 12 showed that in terms of increasing patient MWD, supervised NW was significantly better than unsupervised NW (1001 ± 913m vs 652 ± 373m).**
9. I am very grateful to the reviewer for pointing out my mistake. The conclusion has now been revised, located on lines **469-474** of the manuscript. The revised results are as follows: **Nordic walking can improve the walking ability of PAD patients. However, compared to standard exercise therapy (SET), supervised Nordic walking does not have significant advantages; SET was more effective in enhancing exercise duration. Nordic walking presents a viable option when SET is not available. Further high-quality research is needed due to the limited number of studies to validate these findings.**
10. Thank you very much for the reviewer's suggestions on the format. The margins of Tables 1 and 2 have been adjusted in the manuscript.
11. Finally, due to some modifications in the results section, I also made corresponding changes to the abstract and sensitivity analysis .

The above is my response to the comments raised by the editor and reviewer. Once again, I would like to express my sincere gratitude. The biggest gain during the submission process was the feedback on the manuscript due to insufficient content. Although there are still many shortcomings in my manuscript, I will work hard to make revisions and strive to meet your requirements. Thank you very much.

Looking forward to letters from editors and reviewers, wishing you a happy life!

Yours sincerely.

Zerong Sun, Yongdong Qian
