# Supplementary material for: Effect of Nordic walking on walking ability in patients with peripheral arterial disease: a meta-analysis
Source: PLoS One. 2025 Mar 10;20(3):e0316092. doi: 10.1371/journal.pone.0316092 (PMC11892863; doi:10.1371/journal.pone.0316092)
Supplement: Attachment — Submitted filename: Rebuttal_letter_auresp_3.docx [file pone.0316092.s011.docx]

Rebuttal letter

Dear Editor and Reviewer，

Firstly, I would like to sincerely thank the editor and reviewers for taking the time and effort to review my manuscript, which has given me the opportunity to improve its quality. I am very happy about this. The reviewer provided a lot of valuable suggestions in the view letter, and I carefully read and made revisions to each suggestion. During this process, I learned a lot of knowledge and gained a deeper understanding of the exercise method of Nordic walking. All the parts in the manuscript that have been modified according to the suggestions of the reviewer have been marked in red. Now, I will respond to every point raised by the editor and reviewers.

**Editor's comment：**

1.Thank you for the editor's reminder. I have checked the format of the references and highlighted the changes in red. I have added the month of publication, for example, 2024; 45 (15): 1303-1321 is modified to 2024 Apr14; 45(15):1303-1321.

**Reviewer’s comments:**

1. Thank the reviewer for raising my question. I also realized that there was an error in the footnotes of my forest plot. After reading the meta-analysis you provided, I made changes in the figures, and I also consulted relevant materials. When creating a forest plot with RevMan software, the default research event of the software is "adverse event", such as incidence rate, mortality or negative results. However, if it is a “favorable event”, the default setting of the software needs to be modified. For example, the comprehensive effect size of Figure 5 is -0.06, and the forest plot is slightly biased towards the left of the invalid line, while the result is a favorable event. Therefore, the left footnote should be modified to SET and the right to NW, and the same applies to other graphs. Thank you again to the reviewer for their valuable feedback.
2. Thank you very much for the second reviewer's affirmation of my article.
3. Finally, I only made modifications in the figures and references, and did not make any other changes. Thank you to the editor and reviewers.

The above is my response to the comments raised by the editor and reviewer. Once again, I would like to express my sincere gratitude. The biggest gain during the submission process was the feedback on the manuscript due to insufficient content. Although there are still many shortcomings in my manuscript, I will work hard to make revisions and strive to meet your requirements. Thank you very much.

Looking forward to letters from editors and reviewers, wishing you a happy life!

Yours sincerely.

Zerong Sun, Yongdong Qian
