# Supplementary material for: Effect of Nordic walking on walking ability in patients with peripheral arterial disease: a meta-analysis
Source: PLoS One. 2025 Mar 10;20(3):e0316092. doi: 10.1371/journal.pone.0316092 (PMC11892863; doi:10.1371/journal.pone.0316092)
Supplement: Attachment — Submitted filename: Rebuttal_letter_auresp_4.docx [file pone.0316092.s012.docx]

Rebuttal letter

Dear Editor and Reviewer，

Firstly, I would like to sincerely thank the editor and reviewers for taking the time and effort to review my manuscript, which has given me the opportunity to improve its quality. I am very happy about this. The reviewer provided a lot of valuable suggestions in the view letter, and I carefully read and made revisions to each suggestion. During this process, I learned a lot of knowledge and gained a deeper understanding of the exercise method of Nordic walking. All the parts in the manuscript that have been modified according to the suggestions of the reviewer have been marked in red. Now, I will respond to every point raised by the editor and reviewers.

**Editor's and Reviewer's comment：**

1. Thank you very much to the editor for giving me another opportunity to revise the manuscript. I am very grateful.

Regarding the issue of resolving the forest plot, I carefully checked the footnotes of my forest plot and it should be fine. In the previous revision, I followed the reviewer's suggestion and changed the left side of the forest plot footnote to the control group, which is the SET group, and the right side to the intervention group, which is the NW group. The outcome measure of this study aims to encourage patients to increase their walking distance as much as possible, in order to achieve favorable outcomes. Therefore, the default direction automatically generated by Revman software needs to be modified, and I also referred to forest plots from other journals, as shown in the following figure. If you have any other suggestions, I am happy to make revisions.（The forest plot of resistance training and treadmill walking training is shown below, with an effect size of -0.47, indicating that the results support the treadmill group）。

2. I have revised the format of the references based on the editor's suggestion and highlighted them in red. Additionally, I have replaced reference 16 as the revised article cannot read the entire text.
3. I have uploaded the pictures to PACE and all the pictures meet the requirements.
4. I have added supporting information after the conclusion of the manuscript, and all data in this study are included in the supporting information folder.

The above is my response to the comments raised by the editor and reviewer. Once again, I would like to express my sincere gratitude. The biggest gain during the submission process was the feedback on the manuscript due to insufficient content. Thank you very much.

Looking forward to letters from editors and reviewers, wishing you a happy life!

Yours sincerely.

Zerong Sun, Yongdong Qian
